# Concurrent infection with piscine intestinal coccidia and infectious spleen and kidney necrosis virus associated with mass mortality and severe gastrointestinal pathology in juvenile *Lates calcarifer*

**DOI:** 10.14202/vetworld.2026.1900-1913

**Published:** 2026-05-12

**Authors:** Watcharapol Suyapoh, Sasibha Jantrakajorn, Komkiew Pinpimai, Jakarwan Yostawonkul, Kitipong Angsujinda, Nabhasbhichayabha Daewang, Wanchai Assavalapsakul, Nopadon Pirarat

**Affiliations:** 1 Faculty of Veterinary Science, Prince of Songkla University, Songkhla 90110, Thailand; 2 Aquatic Resources Research Institute, Chulalongkorn University, Bangkok 10330, Thailand; 3 National Nanotechnology Center (NANOTEC), National Science and Technology Development Agency (NSTDA), Pathum Thani 12120, Thailand; 4 Department of Microbiology, Faculty of Science, Chulalongkorn University, Bangkok 10330, Thailand; 5 Department of Pathology, Faculty of Veterinary Science, Chulalongkorn University, Bangkok, Thailand

**Keywords:** aquaculture pathology, co-infection, infectious spleen and kidney necrosis virus, intestinal coccidia, juvenile fish mortality, *Lates calcarifer*, megalocytivirus, piscine parasitology

## Abstract

**Background and Aim:**

Concurrent infections in aquaculture species often exacerbate disease severity and complicate diagnosis and control strategies. This study investigated the pathological and molecular characteristics of co-infection with piscine intestinal coccidia and infectious spleen and kidney necrosis virus (ISKNV) associated with mass mortality in juvenile *Lates calcarifer*.

**Materials and Methods:**

A total of 20 moribund juvenile *L. calcarifer* collected during a mortality outbreak were subjected to necropsy, histopathology, and molecular analyses. Histological examination was performed using hematoxylin and eosin staining. ISKNV infection was confirmed by polymerase chain reaction targeting the major capsid protein (*MCP*) gene, followed by sequencing and phylogenetic analysis. Scanning electron microscopy (SEM) was used to characterize the morphology and ultrastructure of intestinal coccidial oocysts.

**Results:**

All examined fish (100%) were concurrently infected with piscine intestinal coccidia and ISKNV, indicating a strong association with the observed outbreak. Clinically, fish exhibited emaciation, discoloration, and visceral congestion. Histopathology revealed systemic ISKNV infection characterized by basophilic hypertrophic (megalocytic) cells in multiple organs, while coccidia were localized within the intestinal epithelium at epicellular, intracellular, and subepithelial positions. Severe gastrointestinal lesions, including inflammation, epithelial desquamation, and necrosis, were markedly intensified in co-infected tissues. SEM demonstrated rough-surfaced, oval oocysts consistent with piscine intestinal coccidia. Molecular analysis confirmed the presence of ISKNV, with *MCP* gene sequences showing 100% identity with regional and global isolates and clustering within the genotype I lineage. Phylogenetic findings further supported the close evolutionary relationship with strains from Southeast Asia and other regions.

**Conclusion:**

This study provides the first comprehensive evidence of concurrent infection with piscine intestinal coccidia and ISKNV as a significant cause of mass mortality in juvenile *L. calcarifer*. The synergistic pathological effects observed in the gastrointestinal tract highlight the importance of considering polymicrobial infections in disease investigations. Strengthened surveillance, improved biosecurity, and integrated disease management strategies are essential to mitigate the impact of such emerging co-infections in aquaculture systems.

## INTRODUCTION

Asian seabass or barramundi (*Lates calcarifer*) is an economically important fish species in the Asia–Pacific region, particularly in Australia and Southeast Asia [1, 2]. Global fisheries and aquaculture have contributed to this species’ share of worldwide aquatic fish production, which reached 178 million tons between 2000 and 2019 [3]. In recent years, infectious diseases have increasingly caused high mortality in *L. calcarifer* aquaculture. Reported pathogens include bacteria such as Streptococcus agalactiae and S. iniae [4, 5]; gill parasites such as copepods [6] and monogeneans [7, 8]; viruses such as infectious spleen and kidney necrosis virus (ISKNV) [9]; and protozoa such as intestinal coccidia [2, 10]. Chronic piscine intestinal coccidiosis, caused by apicomplexan protozoa including *Goussia* spp., *Eimeria* spp., and *Cryptosporidium* spp. [2, 11–13], is generally considered subclinical but can lead to severe lesions and mortality through immune dysregulation [2, 10, 14]. Impaired host immunity subsequently increases susceptibility to secondary infections and systemic disease [15]. In addition, coccidial infection can alter the gut microbiome, potentially predisposing affected fish to further opportunistic infections [16, 17].

Concomitant infections involving diverse pathogens, including bacteria, fungi, helminths, protozoa, and viruses, are well documented in many fish species [18–20]. In *L. calcarifer*, only a few studies have examined interactions among such pathogens, including parasite–parasite, parasite–bacterium, bacterium–virus, and virus–virus associations, and their synergistic effects on pathology [21–25]. Interactions between intestinal coccidia and viral agents in juvenile *L. calcarifer* have been reported only incidentally, with iridoviral sequences detected in fish concurrently affected by coccidiosis; however, these observations did not demonstrate systemic viral involvement or establish a pathogenic relationship between the two agents [2]. In those reports, viral detection was not associated with characteristic ISKNV-related lesions or enhanced gastrointestinal pathology attributable to co-infection. Megalocytivirus ISKNV remains one of the most significant viral threats to *L. calcarifer* aquaculture worldwide [23, 26–28]. This virus causes significant economic losses in *L. calcarifer* farming, with reported mortality rates of 35–80% during regional outbreaks [26, 29]. ISKNV infection is characterized by systemic dissemination and the formation of megalocytes in multiple organs, including the spleen, kidney, gills, skin, heart, brain, and, notably, the gastrointestinal tract, both as a single infection and in mixed infections [9, 21, 23, 30, 31]. Although ISKNV has been reported in co-infection with other pathogens, including bacterial agents such as Aeromonas hydrophila [32] and viral agents such as nervous necrosis virus (NNV) [33], these reports did not involve concomitant infection with piscine intestinal coccidia, nor did they characterize associated gastrointestinal pathological interactions.

Despite increasing recognition of polymicrobial infections in aquaculture systems, there remains a critical lack of comprehensive studies investigating the interaction between protozoal and viral pathogens in juvenile *L. calcarifer*. Previous reports have primarily focused on single-pathogen infections or limited combinations of pathogens, with minimal attention given to the pathological interplay between intestinal coccidia and ISKNV. Incidental detection of viral sequences in coccidia-infected fish has not been supported by detailed histopathological evidence or confirmation of systemic viral infection. Furthermore, existing studies have not adequately addressed whether co-infection leads to synergistic disease progression, particularly in the gastrointestinal tract, a key site for both pathogen colonization and host immune responses. This knowledge gap limits the understanding of disease dynamics, complicates diagnosis, and hinders the development of effective control strategies in intensive aquaculture systems.

Juvenile stages of *L. calcarifer* are particularly vulnerable to infectious diseases due to ongoing immune development and intensive nursery rearing conditions [34, 35]. Despite the recognized individual pathogenicity of both piscine intestinal coccidia and ISKNV, the pathological consequences of their concurrent occurrence in juvenile *L. calcarifer* remain poorly defined. To our knowledge, based on a structured literature search of indexed databases, no previous study has provided histopathological documentation of simultaneous intestinal coccidiosis and systemic ISKNV infection in *L. calcarifer*. Therefore, the present study aims to characterize this dual-pathogen occurrence in the context of a natural outbreak, with a particular focus on histopathological alterations and organ-specific pathology. By integrating morphological and molecular findings, this study seeks to provide a clearer understanding of mixed infections and their impact on disease severity in seabass aquaculture.

## MATERIALS AND METHODS

### Ethical approval

This study was conducted in accordance with internationally accepted guidelines for the care and use of animals in research and was approved by the Institutional Animal Care and Use Committee of Prince of Songkla University, Thailand (Approval No. MHESI 68014/1731). The study used moribund juvenile *L. calcarifer* submitted for diagnostic investigation during a natural disease outbreak; therefore, no animals were experimentally infected or intentionally sacrificed for research purposes. All procedures were performed in compliance with institutional and national ethical standards for animal welfare. Handling, transportation, and sampling of fish were conducted to minimize stress and suffering, following standard veterinary diagnostic protocols. Necropsy and tissue sampling were performed promptly after collection to ensure specimen integrity while adhering to humane practices. No endangered or protected species were involved in this study, and permission for sample collection was obtained from the aquaculture facility.

### Study period and location

The study was conducted in January 2022 during a mortality outbreak at a commercial *L. calcarifer* nursery farm located in Samut Songkhram Province, central Thailand. Moribund juvenile fish were collected at the outbreak site and transported to the Department of Pathology, Faculty of Veterinary Science, Chulalongkorn University, Bangkok, Thailand, for diagnostic necropsy and histopathological evaluation.

### Study design

This study was designed as a retrospective diagnostic case series investigating a natural outbreak of disease in juvenile *L. calcarifer*. Moribund fish collected during a mortality event were subjected to comprehensive pathological and molecular analyses to characterize concurrent infection with piscine intestinal coccidia and ISKNV. The study combined gross examination, histopathology, molecular detection, sequencing, and ultrastructural analysis to describe pathogen distribution, tissue tropism, and associated lesions. No experimental infection or controlled exposure trials were conducted, and all observations were based on naturally occurring infections.

### Disease history and sample collection

Twenty moribund juvenile *L. calcarifer*, measuring 3–5 cm in total length and exhibiting lethargy followed by sudden death, were collected in January 2022 from nursery tanks at a commercial aquaculture facility in Samut Songkhram Province, central Thailand. The farm reported approximately 90% cumulative mortality during the outbreak period. Fish were reared in shaded ponds under routine aquaculture management conditions, with water salinity maintained at 30 ppt, temperature at 25°C, and pH at 8.2. They were fed a commercial pelleted diet (Profeed 902; Thai Union Feedmill Public Co. Ltd., Samut Sakhon, Thailand) containing 42% crude protein twice daily ad libitum. Moribund fish were submitted for diagnostic evaluation during an outbreak event, and no additional experimental sampling was performed. Specimens were placed in sterile containers within chilled, insulated boxes and transported to the Department of Pathology, Faculty of Veterinary Science, Chulalongkorn University. During transportation, water temperature was maintained at approximately 20–22°C. Necropsy and tissue fixation were performed promptly upon arrival, typically within 2 h of collection. Comprehensive gross and histopathological examinations were conducted. All major organs were inspected for macroscopic lesions. Representative samples of intracoelomic organs were fixed in 10% neutral-buffered formalin for 24 h, processed routinely, embedded in paraffin, sectioned at 5 μm, and stained with hematoxylin and eosin (H&E) according to standard histopathological procedures as described previously [36].

### Identification of ISKNV

Evaluation of megalocytic cells: Evidence of ISKNV infection was initially evaluated using a Nikon ECLIPSE Ni-U advanced upright microscope equipped with a video-capture digital camera (Nikon, Tokyo, Japan). Diagnosis was based on the presence of characteristic lesions, including megalocytic cells, viral inclusion bodies, and basophilic hypertrophic cells in target organs such as the heart, kidney, gas gland, stomach, and other affected tissues [37].

Polymerase chain reaction (PCR) amplification and cloning of the *MCP* gene: Genomic DNA was extracted from the spleen and kidney tissues of *L. calcarifer* using the MasterPure™ Complete DNA & RNA Isolation Kit (Lucigen, Middleton, WI, USA) following the manufacturer’s instructions. DNA concentration and purity (A260/A280 ratio) were determined with a BioPhotometer D30 (Eppendorf AG, Hamburg, Germany). To confirm the presence of ISKNV, the complete ORF of the *MCP* gene was amplified by PCR using a T100™ Thermal Cycler (Bio-Rad Laboratories, Hercules, CA, USA). Each 50 μL reaction contained 1 U Phusion™ High-Fidelity DNA Polymerase (Thermo Fisher Scientific™, Waltham, MA, USA), 1× Phusion HF buffer, 200 μM dNTP mix, 250 nM of each primer, and 200 ng of template DNA. Full-length MCP (1362 bp) was amplified using primers derived from Huang et al. [38], and described by Subramaniam et al. [39], with minor 5′ extensions to improve amplification efficiency. The primer sequences were 5′-GGGGATGTCTGCAATACTCAGGTGC-3′ (forward) and 5′-GGGGTTACAGGATAGGGAAGCCTG-3′ (reverse).

PCR cycling conditions were as follows: initial denaturation at 98°C for 30 s; 35 cycles of 98°C for 10 s, 60°C for 15 s, and 72°C for 45 s; and a final extension at 72°C for 10 min. A no-template control and a positive control were included in each PCR run to monitor contamination and assay performance. The amplicons were separated by electrophoresis on a 1% agarose gel, stained with ethidium bromide, and visualized under ultraviolet illumination using a FireReader V10 Gel Documentation System (UVITEC Ltd., Cambridge, UK). A single band corresponding to the expected MCP ORF size (1,362 bp) was consistently observed. DNA fragments of the expected size were excised from the gel and purified using the Monarch® DNA Gel Extraction Kit (New England Biolabs, Ipswich, MA, USA). Purified products were cloned into the pJET1.2/blunt vector using the CloneJET PCR Cloning Kit (Thermo Fisher Scientific) according to the manufacturer’s instructions and then transformed into *Escherichia coli* DH5α competent cells. Recombinant plasmids were recovered using the Monarch® Plasmid Miniprep Kit (New England Biolabs, Ipswich, MA, USA), and verification and bidirectional Sanger sequencing were performed by Macrogen Inc. (Seoul, Republic of Korea). Sequence alignment with reference ISKNV isolates in GenBank further verified amplification specificity.

### Phylogenetic analysis

The representative nucleotide sequence of the *MCP* gene was analyzed with BioEdit v7.2 and compared with available sequences in the GenBank database using BLASTn to verify nucleotide identity. Multiple sequence alignments and a phylogenetic tree of *MCP* gene sequences from ISKNV isolates across various fish hosts and countries, together with closely related iridoviruses, including red sea bream iridovirus, turbot reddish body iridovirus, and olive flounder iridovirus, were generated using maximum-likelihood analysis with the Kimura 2-parameter model [40]. Rate variation among sites was modeled using a gamma distribution [41], and node support was assessed using 1,000 bootstrap replicates in MEGA [42]. Sequence similarity of the *MCP* gene from the representative isolate was calculated against reference ISKNV sequences retrieved from GenBank using the p-distance model in MEGA software [42].

### Identification of piscine intestinal coccidia

Morphological identification: Histological sections were examined under a Nikon ECLIPSE Ni-U upright microscope equipped with a DS-Fi3 digital camera and NIS-Elements imaging software (Nikon Corporation, Tokyo, Japan). Parasitic stages were identified according to previously published morphological criteria [43, 44]. For descriptive assessment, five non-overlapping high-power fields (×400) were examined in each of three regions of the small intestine (proximal, middle, and distal). Observations included evaluation of parasitic developmental stages and their localization within the intestinal epithelium, including epicellular, intracytoplasmic, and subepithelial positions. A parasitologist confirmed all morphological analyses. The coccidian infection rate was determined.

Morphometric observations using scanning electron microscopy (SEM): Representative intestinal tissues positive for coccidial infection, as confirmed by histopathology, and preserved in 10% neutral-buffered formalin, were selected for SEM. Preparation followed the protocol described previously [45]. Briefly, samples were dehydrated through a graded ethanol series (30%, 50%, 70%, 90%, and 100%), each step lasting 15–30 min, to ensure complete dehydration and prevent structural distortion during critical point drying with liquid CO_2_ using an Autosamdri-931 critical point dryer (Tousimis Research Corporation, Rockville, MD, USA). Dried tissues were mounted on aluminum stubs with double-sided conductive carbon adhesive tape and coated with a 25 nm layer of gold using an SPI-Module sputter coater (SPI Supplies, West Chester, PA, USA) to prevent charging under the electron beam. Surface ultrastructural features of the coccidia were examined with a field-emission scanning electron microscope (Apreo FE-SEM; Thermo Fisher Scientific™, Eindhoven, Netherlands) operated in high-vacuum mode at 5–15 kV. Digital micrographs at multiple magnifications documented detailed coccidial morphology. Oocyst dimensions (length × width) were determined from calibrated digital micrographs using ImageJ software (National Institutes of Health, Bethesda, MD, USA). For each sample, 6–10 oocysts were measured, and values were expressed as mean ± standard deviation.

### Histopathology of concomitant infection

Intracoelomic organs, including the heart, kidney, gas gland, stomach, bulbus arteriosus, and intestine, were fixed in 10% neutral-buffered formalin for 24 h. After fixation, tissues were dehydrated through a graded ethanol series, cleared in xylene, and embedded in paraffin wax according to routine histological procedures. Paraffin blocks were sectioned at 5 μm thickness using a rotary microtome, mounted on glass slides, and stained with H&E following previously described protocols [36]. Histological changes were evaluated by veterinary pathologists with attention to the distribution and severity of inflammatory cell infiltration, tissue degeneration, necrosis, vascular alterations, and the presence of characteristic ISKNV-associated megalocytic cells and coccidian developmental stages. The presence, extent, and pattern of these lesions were recorded to assess the impact of dual infection on organ pathology and to relate microscopic findings to the observed high mortality in juvenile *L. calcarifer*.

### Statistical analysis

All quantitative data are presented as descriptive statistics. Oocyst measurements obtained from SEM were expressed as mean ± standard deviation. No inferential statistical analyses were performed, as the study was designed as a retrospective diagnostic case series without predefined comparative groups or hypothesis-driven testing. Data processing and calculation of descriptive statistics were performed using Microsoft Excel (Microsoft Corporation, Redmond, WA, USA).

## RESULTS

### Disease history in juvenile *L. calcarifer*

In January 2022, farms in central Thailand reported a severe mortality event affecting early juvenile *L. calcarifer*, with cumulative losses estimated at approximately 90% of the stock. The fish originated from broodstock maintained in closed earthen ponds. Fertilized eggs were transferred to hatching tanks supplied with brackish water, and the resulting larvae were reared in nursery tanks until the juvenile stage. During rearing, the fish became lethargic and died suddenly without exhibiting specific external signs of disease. Farmers routinely used prophylactic antibiotics to prevent bacterial infections and enhance productivity; however, mortalities persisted, suggesting that bacteria were not the primary cause. These observations indicated that other pathogens, such as parasites or viruses, were likely responsible for the outbreak.

### Gross pathological findings at necropsy

Fish carcasses were placed in sterile containers within large, chilled plastic buckets and transported to the Department of Pathology, Faculty of Veterinary Science, Chulalongkorn University, for necropsy. Each specimen was weighed, measured, and examined for gross pathological changes. Affected juvenile *L. calcarifer* typically displayed grayish-black body discoloration and marked emaciation. No obvious external lesions such as necrosis, lepidorthosis, cloudy eyes, hemorrhage, or ulcers were detected. Congestion of intracoelomic organs, particularly the intestine, liver, kidney, and spleen, was consistently observed. Based on these external and internal findings, bacterial disease was considered unlikely, suggesting that viral or parasitic pathogens were the primary causes of mortality.

### Infection of piscine intestinal coccidia

**Figure 1:**
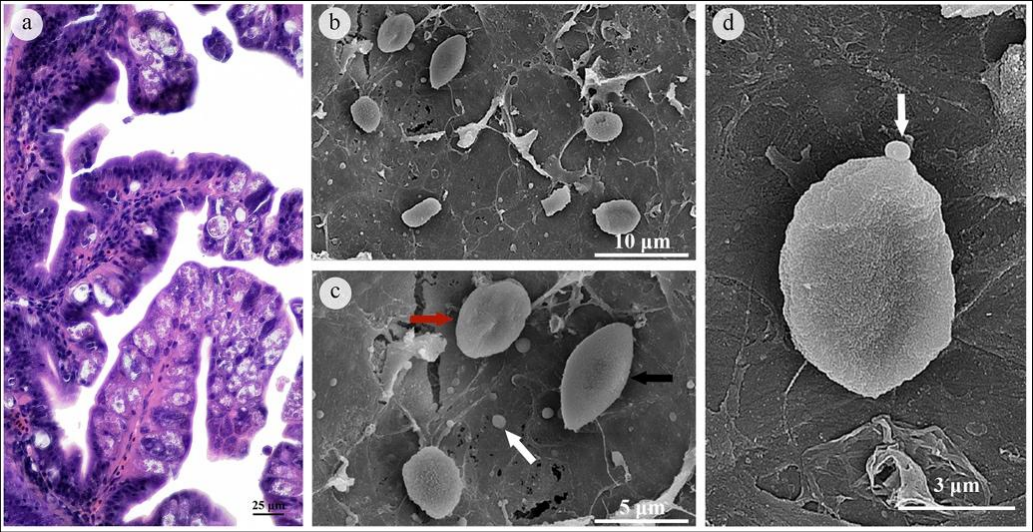
Morphological characteristics of piscine intestinal coccidia in juvenile Asian seabass (*Lates calcarifer*). (A) Histological section of the intestine showing different developmental stages of piscine intestinal coccidia within the mucosa and submucosa, including meronts, gamonts, and oocysts. The parasites are observed in epicellular, intracellular, and subepithelial locations. (B–D) Scanning electron micrographs of oocysts demonstrating rough surface morphology without a polar cap or micropyle.

To assess possible parasitic involvement in the mortality event, gastrointestinal tissues, including the stomach, pyloric ceca, and intestine, were examined microscopically for the presence of piscine intestinal coccidia. All 20 fish (100%) were positive for coccidial infection. Histological identification was based on morphological characteristics of the parasite, which occupied epicellular, intracellular, and subepithelial locations within the mucosa and submucosa (Figure 1a). Three major developmental stages were observed: meronts, gamonts, and oocysts.

Oocysts were typically oval and frequently aggregated within intracellular or subepithelial positions of the pyloric cecum and intestine. Most were unsporulated and surrounded by a thin oocyst wall. Gamonts, which develop from merozoites, differentiate into macrogamonts and microgamonts. Macrogamonts were irregular to oval, with prominent central nuclei and distinct eosinophilic peripheral bodies, whereas microgamonts were spherical to oval and contained multiple peripheral dark basophilic nuclei. Both gamont types were most often located at epicellular positions, admixed with abundant mucus and cellular debris. Some macrogamonts were embedded in the intestinal epithelium and aggregated into small clusters.

Because caps or micropyles of oocysts are not discernible by light microscopy, SEM was employed to characterize surface ultrastructure. SEM revealed rough-surfaced oocysts lacking both a polar cap and a micropyle (Figures 1b–d). The mean oocyst size was 5.18 ± 1.20 μm in length and 3.38 ± 0.59 μm in width (n = 6), with observed ranges of 3.8–6.9 μm in length and 2.6–4.1 μm in width. These dimensions fall within the reported size range of small- to medium-sized piscine intestinal coccidia described in previous studies, although definitive genus-level assignment cannot be established based solely on morphology.

### ISKNV infection and histopathological characteristics

**Figure 2:**
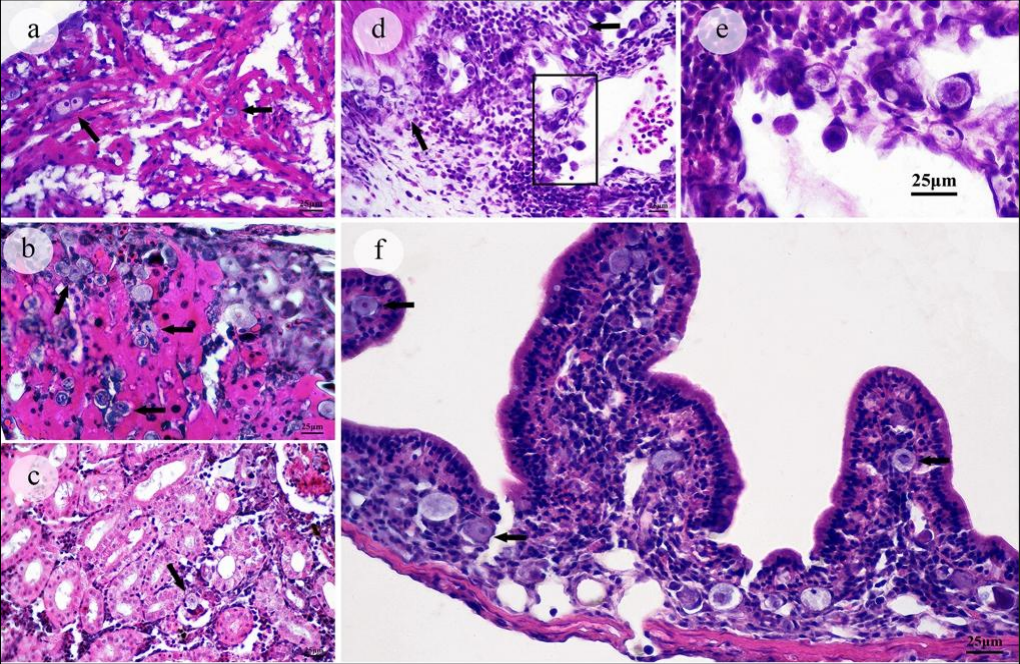
Histopathological features of infectious spleen and kidney necrosis virus infection in juvenile Asian seabass (*Lates calcarifer*). (A–E) Presence of megalocytic cells in various organs, including the heart, kidney, bulbus arteriosus, and gas gland. These cells exhibit hypertrophy, basophilic inclusion bodies, enlarged nuclei, and a high nucleus-to-cytoplasm ratio. (F) Intestinal tissue showing concurrent coccidial infection with marked lymphocytic infiltration and numerous megalocytic cells.

ISKNV infection was further investigated in juvenile *L. calcarifer*. Histopathological identification was based on the presence of megalocytic or basophilic hypertrophic cells containing inclusion bodies scattered throughout the parenchyma of various coelomic organs. Microscopic examination revealed numerous hypertrophic cells in the heart, kidney, bulbus arteriosus, submucosal vessels, and gas gland (Figures 2a–f). These cells exhibited a high nucleus-to-cytoplasm ratio, a centrally located prominent nucleolus, enlarged nuclei with peripherally distributed chromatin, and distinct intracytoplasmic basophilic inclusions.

Consistently, the stomach and intestinal tissues already affected by coccidiosis exhibited marked lymphocytic infiltration with numerous megalocytic cells (Figure 2f). In contrast, no significant lesions were detected in the spleen, liver, gills, or brain.

**Figure 3:**
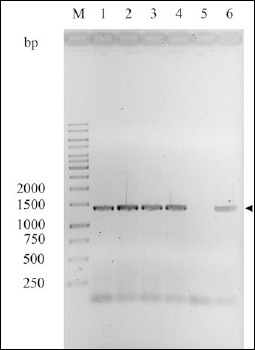
Detection of infectious spleen and kidney necrosis virus based on major capsid protein gene amplification. Polymerase chain reaction amplification of the major capsid protein gene showed a specific band at approximately 1,500 base pairs in kidney and spleen samples. Positive and negative controls confirm assay specificity and absence of contamination.

To confirm the viral etiology, molecular detection targeting the ORF of the MCP gene was performed. PCR amplification of kidney and spleen samples produced an approximately 1.4 kb fragment (Figure 3). The amplicons were cloned into a vector, transformed into *E. coli*, and sequenced. Sequence analysis confirmed the presence of a 1,362-nucleotide MCP ORF, thereby verifying ISKNV infection.

### Sequencing and phylogenetic analysis of ISKNV

All juvenile *L. calcarifer* samples tested positive for ISKNV (Figure 3). Molecular identification was conducted by PCR amplification and sequencing of the *MCP* gene. Pairwise identity matrix analysis of *MCP* gene sequences revealed that isolate CU-1 was 100% identical to ON743043 (China), AF370008 (China), AB666337 (Malaysia), PV101205 (Singapore), and PQ351746 (Thailand). The next closest match was MZ152127 (India), sharing 99.70% similarity with CU-1 (Table 1). The representative nucleotide sequence obtained in this study has been deposited in GenBank under accession number PX442651.

**Table 1:** Pairwise identity matrix of reference isolates and the ISKNV isolate based on *MCP* gene sequences.

Isolate	ON743043	MZ152127	MT642065	KY440045	KY440041	AF370008	AB666337	PV101205	PQ351746	PX442651 (CU-1)
ON743043	–									
MZ152127	99.70	–								
MT642065	98.80	98.50	–							
KY440045	98.70	98.40	99.90	–						
KY440041	98.70	98.40	99.90	100.00	–					
AF370008	100.00	99.70	98.80	98.70	98.70	–				
AB666337	100.00	99.70	98.80	98.70	98.70	100.00	–			
PV101205	100.00	99.70	98.80	98.70	98.70	100.00	100.00	–		
PQ351746	100.00	99.70	98.80	98.70	98.70	100.00	100.00	100.00	–	
PX442651 (CU-1)	100.00	99.70	98.80	98.70	98.70	100.00	100.00	100.00	100.00	–

ISKNV = Infectious spleen and kidney necrosis virus, MCP = Major capsid protein, CU-1 = Chulalongkorn University isolate 1. Blank cells in the upper triangle indicate redundant pairwise comparisons and were intentionally omitted.

**Figure 4:**
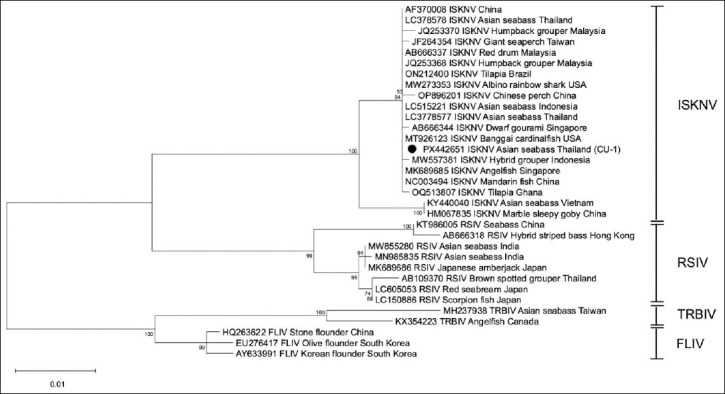
Phylogenetic relationship of infectious spleen and kidney necrosis virus isolates based on major capsid protein gene sequences. Phylogenetic tree constructed using the maximum likelihood method showing clustering of the study isolate with reference isolates from different geographic regions. The tree demonstrates distinct clades corresponding to infectious spleen and kidney necrosis virus and related iridoviruses. Bootstrap values are indicated at branch nodes.

Phylogenetic analysis of *MCP* gene sequences demonstrated that ISKNV, red sea bream iridovirus, turbot reddish body iridovirus, and olive flounder iridovirus each form distinct, well-supported monophyletic clades, consistent with their classification as separate but closely related megalocytiviruses. The majority of ISKNV isolates clustered into a strongly supported monophyletic group (99% bootstrap). Within this group, bootstrap support for internal subclades varied; however, the CU-1 isolate in this study clustered with a broad range of hosts, including humpback grouper (Malaysia), albino rainbow shark (USA), mandarin fish (China), hybrid grouper (Indonesia), Banggai cardinalfish (USA), dwarf gourami (Singapore), tilapia (Brazil), and *L. calcarifer* (Thailand and Indonesia), with strong support (99% bootstrap), confirming its identity as ISKNV. Notably, this isolate was clearly distinct from ISKNV previously reported in *L. calcarifer* from Vietnam (KY440040) and in marble sleepy goby from China (HM067835) (Figure 4).

### Histopathological effects of co-infection

**Figure 5:**
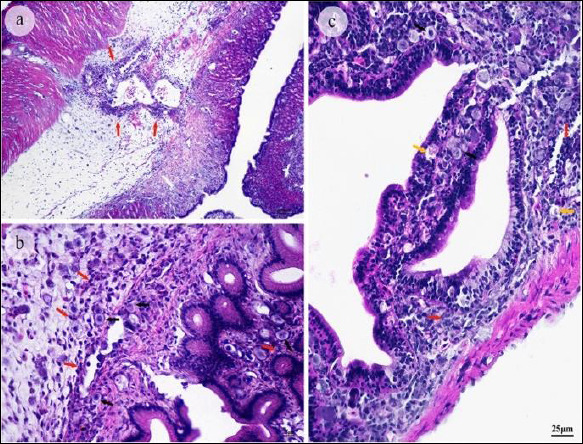
Histopathological changes in the gastrointestinal tract of naturally co-infected juvenile Asian seabass. (A) Mononuclear cells and granulocytes (red arrows) are infiltrating connective tissue and perivascular areas of the submucosa in the stomach. (B) Basophilic hypertrophic (megalocytic) cells (black arrow) surrounded by dense inflammatory infiltrates of mononuclear cells and granulocytes (red arrows) at higher magnification. (C) Intestinal co-infection with coccidia (yellow arrow) and ISKNV (black arrow), accompanied by intense mononuclear and granulocytic infiltration. Associated lesions included intestinal inflammation, vascular congestion, intraepithelial lymphocyte infiltration, epithelial desquamation, and tissue necrosis.

To evaluate the pathological impact of concurrent piscine intestinal coccidia and ISKNV infection, detailed histopathological examinations were performed on all submitted fish. Overall, ISKNV infection was characterized by hypertrophic (megalocytic) cells accompanied by variable degrees of inflammation and necrosis. Minimal lymphocytic infiltration was observed around hypertrophied cells in organs not affected by coccidia, including the heart, kidney, bulbus arteriosus, and gas gland (Figures 5a–c).

In contrast, tissues exhibiting dual infection, notably the stomach and intestine, showed more extensive inflammatory lesions where both pathogens were present (Figures 5a–c), which were associated with moderate to severe inflammation, mucosal injury, and cellular necrosis.

In the stomach, inflammatory cell infiltration extended through the mucosa, submucosa, and muscularis. The infiltrate was composed primarily of mononuclear cells (macrophages and lymphocytes), with granulocytes present in lesser numbers. Inflammation typically surrounds viral lesions, which are marked by basophilic hypertrophic cells. Moderate to large numbers of inflammatory cells were concentrated in the mucosa and submucosa, particularly around the vascular endothelium, in perivascular areas, and around epithelial cells. Fewer macrophages and lymphocytes were found in the muscular layer and gut wall, whereas granulocytes were more abundant in the mucosal and submucosal zones. Gastric pathology included necrosis, vascular congestion and hemorrhage, edema, degeneration of the muscular layer, and mucosal and submucosal fibrosis (Figures 5a and b). Necrosis, characterized by nuclear and cell membrane disruption, was associated with moderate-to-severe inflammation. Marked congestion or hyperemia, caused by dilation of blood vessels with erythrocyte accumulation, was common in areas with active leukocytic infiltration. In severe cases, inflammation extended into the muscular layer and the gastric wall, resulting in myocyte degeneration and muscle edema. Chronic lesions were accompanied by mucosal and submucosal fibrosis.

Inflammation in the intestine was more severe than in the stomach. The infiltrate consisted predominantly of macrophages and lymphocytes, with occasional granulocytes in the lamina propria. Lesions initially appeared around the submucosa, where both coccidia and ISKNV were present (Figure 5c), and extended into the epithelium and muscular layers. Severe inflammatory changes correlated with epithelial desquamation, villous denudation, congestion, nuclear centralization within enterocytes, and widespread tissue necrosis.

These findings demonstrate concurrent infection of piscine intestinal coccidia and ISKNV-associated with marked gastrointestinal pathology, characterized by extensive mucosal damage in juvenile *L. calcarifer*.

## DISCUSSION

### Piscine intestinal coccidia infection and pathological implications

Single infections with piscine intestinal coccidia, apicomplexan protozoa that parasitize the gastrointestinal tract, represent a significant health concern for juvenile fish in Southeast Asia and are frequently associated with diverse intestinal pathologies [2, 10, 46]. Most coccidian species display strict host specificity and are typically reported as single infections in various fish species [12, 47, 48]. Although mixed infections have been described, such as coccidia–bacteria [49], coccidia–helminth [25], and coccidia–coccidia combinations including mixed Goussia spp. [44], mixed Cryptosporidium spp. [50], and mixed Eimeria spp. [51], co-infection of coccidia with major pathogenic viruses has not been described previously.

The present study provides the first evidence of concomitant infection of piscine intestinal coccidia with a pathogenic virus, ISKNV, in juvenile *L. calcarifer*. We documented the general infection status, performed phylogenetic analysis, and demonstrated concurrent infection associated with pronounced gastrointestinal lesions observed on histopathological examination. A high proportion of dual infections was observed in the intestinal tract, consistent with previous reports of coccidial [10, 14, 44] and viral infections [9]. The marked susceptibility of juvenile fish suggests an immature or compromised immune system, which reduces host defenses against both protozoal and viral pathogens [52, 53].

Piscine intestinal coccidia were identified in the intestinal tract of *L. calcarifer* through light microscopic examination and SEM-based morphological assessment. The measured oocyst dimensions were 5.18 ± 1.20 μm in length and 3.38 ± 0.59 μm in width (range 3.8–6.9 × 2.6–4.1 μm). These measurements are inconsistent with *Cryptosporidium* spp., which are typically considerably smaller [25, 48]. Based on size, the observed parasites are more compatible with members of the larger-bodied coccidian genera reported in fish, including *Eimeria* spp., *Goussia* spp., *Epieimeria*, *Calyptospora*, and *Crystallospora*, as described in previous studies [44, 54, 55]. Considering the anatomical location of infection (intestinal epithelium), epicellular development, and the predominantly oval rather than spherical oocyst morphology, the findings are less consistent with Calyptospora and *Crystallospora* spp., which are generally described as spherical and have distinct morphological characteristics [56].

Among the remaining candidates, the morphometric profile and site of infection are most compatible with Eimeria-like or Goussia-like piscine intestinal coccidia [54, 55, 57]. However, due to overlapping morphological characteristics among piscine coccidian genera, definitive genus- or species-level identification cannot be established without molecular confirmation, and this taxonomic limitation should be considered when interpreting the findings. Infection with piscine intestinal coccidia, including *Goussia* spp. and *Eimeria* spp., has been reported to disrupt intestinal homeostasis [44], alter the gut microbiota, and increase susceptibility to secondary pathogens in fish, potentially predisposing affected individuals to disease or mortality [16, 17]. Previous reports from Thai aquatic environments have documented *Eimeria* spp. and *Cryptosporidium* spp. in various fish hosts [24, 25, 48], highlighting the diversity of piscine intestinal coccidia in the region. These findings suggest that the diversity and distribution of piscine intestinal coccidia in Thailand may be broader than currently recognized and remain incompletely characterized.

### ISKNV infection and phylogenetic characterization

ISKNV was detected in diseased juvenile *L. calcarifer* by both histopathological and molecular methods. Megalocytic cells containing basophilic inclusions were systemically distributed across multiple coelomic organs, consistent with previous descriptions in *L. calcarifer* aquaculture [26, 30]. Phylogenetic analysis of the *MCP* gene revealed that the *L. calcarifer* ISKNV isolates obtained in this study clustered firmly within the established ISKNV lineage. This placement confirms their phylogenetic identity and evolutionary relatedness to previously reported isolates (genotype I ISKNV) from Asia, particularly those from Thailand, Malaysia, and Indonesia [26, 58].

Notably, this cluster was clearly distinct from genotype II ISKNV, which has been reported from Vietnam [27]. ISKNV has been widely reported in *L. calcarifer* and is recognized as a major viral pathogen in marine aquaculture. Previous studies have documented co-infections involving ISKNV and other viral pathogens, such as red-spotted grouper nervous necrosis virus, associated with increased mortality in juvenile *L. calcarifer* [33]. However, to our knowledge, concurrent infection of ISKNV with piscine intestinal coccidia has not been documented in this host species.

### Pathological interaction and implications of co-infection

This study documents a previously unreported concurrent infection of piscine intestinal coccidia and ISKNV-associated with mass mortality in juvenile *L. calcarifer* in Thailand. Histopathological examination revealed classical systemic ISKNV-associated megalocytic changes in multiple visceral organs, accompanied by extensive necrotizing enteritis localized to the intestine, the primary site of coccidian infection. Extra-intestinal changes, including mild to moderate gastric perivasculitis, myocarditis, aerocystitis, and tubulointerstitial nephritis, were comparatively less severe.

In previously described ISKNV infections in *L. calcarifer*, systemic dissemination with prominent megalocytic cells in the spleen and kidney represents the dominant pathological pattern, whereas severe necrotizing enteritis is not consistently emphasized as a major feature [30]. In contrast, isolated intestinal coccidiosis is typically characterized by epithelial disruption and localized mucosal inflammation without systemic viral-type pathology [10, 24, 59, 60]. The combined distribution of systemic viral lesions and pronounced intestinal necrosis observed in the present study, therefore, differs from the typical presentation of either condition alone and supports the interpretation of concurrent infection.

Previous studies have shown that intestinal coccidia preferentially infect specific intestinal segments in juvenile *L. calcarifer*, particularly the anterior portion, where localized tissue damage and inflammatory responses may compromise mucosal integrity [10]. Experimental and observational studies in fish have also suggested that piscine intestinal coccidia can disrupt intestinal homeostasis, alter the gut microbial community [16], and induce oxidative stress responses, including the production of reactive oxygen species leading to epithelial damage and apoptosis [61]. Such alterations may be associated with reduced host resilience and increased susceptibility to secondary infections.

In the present study, however, no immune gene expression analysis, cytokine profiling, microbiome assessment, or experimental challenge trials were performed. Therefore, any proposed interactions between piscine intestinal coccidia and ISKNV remain speculative and should be interpreted with caution. In addition to these limitations, this investigation was conducted as a retrospective outbreak-based study. Quantitative lesion scoring, statistical comparisons, quantitative viral load assessment (including real-time PCR), and parasitic intensity measurements were not performed. Only moribund fish from a single outbreak were examined, and no healthy or single-pathogen control groups were available, limiting causal interpretation.

In addition, molecular characterization of piscine intestinal coccidia and bacteriological examination were not undertaken. Accordingly, the findings should be interpreted as descriptive evidence of concurrent infection associated with marked gastrointestinal pathology rather than proof of direct pathogen interaction or causation. Further controlled experimental studies are warranted, including challenge trials in juvenile *L. calcarifer* using molecularly characterized ISKNV isolates and intestinal coccidia, with sequential or simultaneous infection designs to assess lesion progression, pathogen load, and host immune responses.

## CONCLUSION

This study demonstrates, for the first time, concurrent infection of piscine intestinal coccidia and ISKNV-associated with mass mortality in juvenile *L. calcarifer*. All examined fish were positive for both pathogens, indicating a 100% co-infection rate during the outbreak. Histopathological findings revealed systemic ISKNV infection characterized by megalocytic cells across multiple organs, along with severe gastrointestinal lesions, including epithelial desquamation, inflammation, and necrosis, associated with coccidial infection. Ultrastructural observations further confirmed the presence of coccidia with characteristic oocyst morphology, and molecular analysis of the MCP gene verified ISKNV identity and its close phylogenetic relationship with regional and global isolates. These findings collectively highlight the pronounced pathological burden associated with dual infection, particularly within the gastrointestinal tract.

From a practical perspective, the results emphasize the importance of considering polymicrobial infections in routine disease diagnosis and management in aquaculture systems. Reliance on single-pathogen diagnostics may lead to underestimation of disease complexity and inappropriate control strategies. Integrated surveillance approaches combining histopathology, molecular diagnostics, and parasitological assessment are essential for accurate detection of co-infections. Strengthening biosecurity measures, improving water quality management, and reducing stress in intensive rearing systems are critical to minimizing susceptibility to such infections. Early detection programs and targeted health monitoring of juvenile stages may significantly reduce economic losses in seabass aquaculture.

A major strength of this study lies in its comprehensive diagnostic approach, integrating histopathology, ultrastructural analysis, and molecular confirmation to characterize co-infection under natural outbreak conditions. The study provides novel evidence linking intestinal coccidiosis with systemic ISKNV infection and expands current understanding of disease interactions in aquaculture species. However, several limitations should be acknowledged. The study was retrospective and based on a single outbreak, with no healthy or single-pathogen control groups available for comparison. Quantitative assessments, including pathogen load, lesion scoring, and host immune responses, were not performed. In addition, molecular identification of piscine intestinal coccidia and bacteriological analyses were not conducted, limiting species-level resolution and exclusion of additional co-infecting agents.

Future research should focus on controlled experimental studies to elucidate the mechanistic interactions between piscine intestinal coccidia and ISKNV, including sequential and simultaneous infection models in juvenile *L. calcarifer*. Investigations incorporating immune gene expression, microbiome profiling, and quantitative pathogen dynamics are necessary to better understand host–pathogen interactions. Development of preventive strategies, including improved hatchery management, targeted therapeutics, and potential vaccine approaches against ISKNV, should also be prioritized.

In conclusion, concurrent infection with piscine intestinal coccidia and ISKNV represents an emerging and significant threat to juvenile *L. calcarifer* aquaculture. Recognition of such co-infections is essential for accurate diagnosis, effective disease management, and sustainable aquaculture production.

## DATA AVAILABILITY

The data generated during the study are included in the manuscript.

## AUTHORS’ CONTRIBUTIONS

WS: Conceptualization, formal analysis, investigation, methodology, validation, writing – original draft, and writing – review and editing. SJ: Methodology, validation, and writing – original draft. KP: Formal analysis and methodology. JY: Methodology and writing – review and editing. KA: Methodology, validation, and writing – review and editing. ND: Methodology. WA: Investigation, methodology, and validation. NP: Conceptualization, formal analysis, funding acquisition, investigation, methodology, resources, validation, writing – original draft, and writing – review and editing. All authors have read and approved the final version of the manuscript.
